# Circulating 25-Hydroxyvitamin D Levels in Fully Breastfed Infants on Oral Vitamin D Supplementation

**DOI:** 10.1155/2010/235035

**Published:** 2009-12-09

**Authors:** Carol L. Wagner, Cindy Howard, Thomas C. Hulsey, Ruth A. Lawrence, Sarah N. Taylor, Heather Will, Myla Ebeling, Jay Hutson, Bruce W. Hollis

**Affiliations:** ^1^Department of Pediatrics, Medical University of South Carolina, Charleston, SC 29425, USA; ^2^Department of Pediatrics, Rochester General Hospital, Rochester, NY 14621, USA; ^3^Golisano Children's Hospital, University of Rochester Medical Center, Rochester, NY 14642, USA

## Abstract

*Objective*. To examine the effectiveness of oral vitamin D_3_ (400 IU) supplementation on the nutritional vitamin D status of breastfeeding infants. *Design*. As part of a larger ongoing vitamin D RCT trial of lactating women, infants of mothers assigned to control received 1 drop of 400 IU vitamin D_3_/day starting at one month of age. Infant 25(OH)D levels (mean ± S.D.) were measured by RIA at visits 1, 4, and 7. *Results*. The infant mean ± S.D. 25(OH)D at baseline was 16.0 ±9.3 ng/mL (range 1.0–40.8; *n* = 33); 24 (72.7%) had baseline levels <20 ng/mL (consistent with deficiency). The mean levels increased to 43.6 ±14.1 (range 18.2–69.7) at 4 months and remained relatively unchanged at month 7: 42.5 ±12.1 ng/mL (range 18.9–67.2). The change in values between 1 and 4 months and 1 and 7 months was statistically significant (*P* ≤ .0001), and despite a decrease in dose per kilogram, values were not significantly different between months 4 and 7 (*P* = .66). *Conclusions*. Oral vitamin D_3_ supplementation as an oil emulsion was associated with significant and sustained increases in 25(OH)D from baseline in fully breastfeeding infants through 7 months.

## 1. Introduction

Among breastfed infants, the risk of vitamin D deficiency is significant [1, 2]. Human milk, though replete in most nutrients, is sorely lacking in vitamin D at the current maternal intake recommendation of 400 IU/day. Human milk's total vitamin D or its antirachitic activity obtained from mothers receiving 400 IU/day typically contains 33–68 IU/L, far below the estimated daily infant requirement of 400 IU/L [3–5], the necessary level for the prevention of rickets [1]. Therefore, oral supplementation or sufficient sun exposure is necessary to ensure adequate vitamin D status for proper calcium absorption and bone mineralization [3–6].Recent research has also linked vitamin D to innate immunity [7–9]. Furthermore, vitamin D deficiency has been associated with a number of disease states including but certainly not limited to rheumatoid arthritis [10], multiple sclerosis [11–14], type I and II diabetes [15–19], Crohn's disease [20], cardiovascular disease [21, 22], and a number of cancers [23, 24]. With this knowledge, it is clear that vitamin D supplementation of the breastfedinfant is necessary and recommended by the American Academy of Pediatrics and the Canadian Pediatrics Society [5, 25].

The revised statement by the American Academy of Pediatrics [26] brings forth the question of whether or not vitamin D-only preparations are as effective as the time-honored use of infant multivitamin preparations in achieving adequate vitamin D status in breastfed infants. In the past, only multivitamin preparations were available for dispensing to infants; however, this resulted in breastfed infants being given other vitamins that were not, for the vast majority, necessary. During recent years, vitamin D-only preparations have become available, some of which are aqueous and others which are oil-based preparations.

As part of an ongoing vitamin D supplementation trial of fully lactating women and their infants, we sought to determine whether a dosing regimen that utilizes an oil emulsion of one drop per day to deliver 400 IU/day is effective in raising infant 25(OH)D levels from baseline. We hypothesized that one drop per day dosing regimen would be easier to administer than other preparations as the drop could be placed on a finger and then applied to the infant's tongue or mouth or on the mother's nipple just prior to suckling. While the parent randomized clinical trial will not be completed until 2011, it is essential to report preliminary data on the effectiveness of one drop per day dosing of vitamin D to aid clinicians in their decision-making process concerning vitamin D preparations for infants. We report the findings of 33 infants who completed the study and who were given 400 IU vitamin D_3_/day in a blinded fashion.

## 2. Methods

### 2.1. Subjects

Approval for this study was granted by the Institutional Review Boards for Human Subjects of the Medical University of South Carolina (MUSC), HR number 16536; and the University of Rochester (U of R), HR number RSRB00014460; and the General Clinical Research Centers of MUSC (GCRC; Protocol number 752) and U of R (Protocol GCRC; Protocol number 1129 ). Fully lactating mothers [27] within 4–6 weeks' postpartum were eligible for inclusion in the study if they planned to continue full breastfeeding for the next six months. Following their written, informed consent, the subjects were randomly divided into three groups at each study site. Exclusion criteria included preexisting type I or type II diabetes, hypertension, parathyroid disease, and uncontrolled thyroid disease. Subjects were compensated for their participation with gift cards given at the end of each visit.

### 2.2. Study Design

This was part of an ongoing randomized, double-blind, and placebo control trial of lactating mothers and their infants. Following written informed consent, mothers were randomized to one of three vitamin D supplementation regimens: Group 1: 400 IU vitamin D_3_/day (0 IU vitamin D_3_—placebo and 1 prenatal vitamin containing 400 IU vitamin D_3_), Group 2: 2,400 IU vitamin D_3_/day (2,000 IU vitamin D_3_ and 1 prenatal vitamin containing 400 IU vitamin D_3_), or Group 3: 6,400 IU vitamin D_3_/day (6,000 IU vitamin D_3_ and 1 prenatal vitamin containing 400 IU vitamin D_3_). Only the results of the infants randomized to the control group are reported here.

The mothers also were provided in a blinded fashion with a liquid supplement with the instructions to give one drop per day to their nursed infants. Mothers in Group 1 gave their infants one drop (400 IU vitamin D_3_) per day of a commercially available oil emulsion (Bio-D-Mulsion, Biotics Research Laboratories, Rosenberg, TX). The vitamin D status of those infants in the control group who received 400 IU/day vitamin D_3_ was measured by circulating 25(OH)D at 1, 4, and 7 months of age. Study participants and research staff have remained blinded to the assignment group throughout the study.

### 2.3. Use of Formula

Because of the potential confounder of proprietary formulas that contain 400 IU/L vitamin D, each mother was given up to six 4-oz bottles per month of vitamin D-free formula with the instructions to avoid use of any formula but if there was a need to supplement, the vitamin D-free formula prepared by Mead-Johnson, Evansville, IN was used. Each mother was to record any vitamin D-free and/or regular formulas use during the study period. Therefore, the dietary source of vitamin D in these infants came from the vitamin D supplement provided.

### 2.4. Sunlight Exposure

A diary of sunlight exposure of the mother and her infant was kept. In order to quantify any possible sunlight exposure contributing to endogenous generation of vitamin D_3_, degrees of skin pigmentation of the underarm, and forearm were recorded at each visit.

### 2.5. Degree of Skin Pigmentation

Skin pigmentation changes were monitored monthly in mother and infant using the SmartProbe 400 (IMS, Inc., Milford, CT), a spectrophotometer device that measures degrees of pigmentation on a continuous scale from 0 to 100, 0 being absolute black and 100 being absolute white. Each mother had pigmentation measurements recorded from her exposed forearm, underarm, and stomach, with 2 readings averaged and recorded. Each infant had pigmentation measurements recorded from the forearm and upper thigh. Of note, mothers were instructed to use sunscreen if outdoors for more than 15 minutes and to avoid direct sunlight exposure of their infants during the first six months.

The difference between forearm and underarm is an indirect measure of sunlight exposure or tanning and was tracked over time. Use of a spectrophotometer has been demonstrated to be effective as an objective, reproducible measurement in determinations of sunlight exposure [28].The spectrophotometer allows measurement of color changes in the skin specifically due to UV radiation [29].

### 2.6. Season

Mothers and their infants were enrolled throughout the year. The date and season of each visit were recorded. The seasons were defined as Spring (March 21–June 20), Summer (June 21–September 20), Fall (September 21–December 20), and Winter (December 21–March 20). Sunny months at latitude 32°N based on ultraviolet light indices occur from April through October. Relative non-sunny months comparatively occur from November through March. At latitude 43°N, sunny months occur May through mid-September and relative non-sunny months occur from mid-September through April.

### 2.7. Laboratory Measurements

For this component of the study, circulating 25(OH)D_3_ was measured in ng/mL in infant blood samples by established methods [30] in the laboratory of Dr. Bruce Hollis, MUSC.

### 2.8. Maintaining Study Blindness

To maintain study staff and investigator blinding, the data coordinating center provided the data to the study investigators and the Data Safety & Monitoring Committee in a blinded manner.

### 2.9. Statistical Methods

The primary analysis was to examine the changes in 25(OH)D between the three time periods and secondarily to determine whether the season when the sample was obtained was associated with any changes in 25(OH)D [31]. 25(OH)D, the primary outcome variable, was expressed as the mean ± S.D. and the range for the group at each of the three time points. Paired Student's *t*-test and repeated measures ANOVA were performed on the data. In addition, a repeated measure ANOVA was performed on the data at the three time points. Significance was set at .05 a priori.

## 3. Results

In this ongoing vitamin D supplementation trial of lactating women and their infants, 54 were enrolled in the study and were randomized to the control group in a blinded fashion. Of the 54 women and their infants, 33 have completed the study through visit 7, 39 have completed through visit 4, and 15 had stopped participation between visits 1 and 4, due to cessation of breastfeeding.

**Figure 1:**
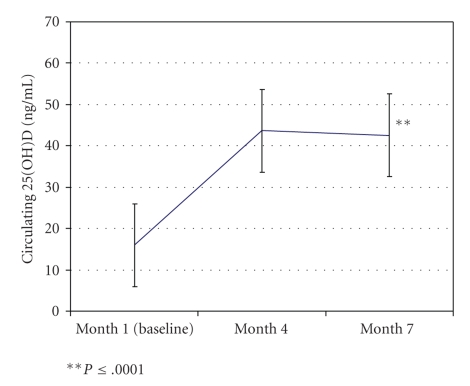


As shown in Table 1 and Figure 1, analyzing all values per study visits 1, 4, and 7, there was a significant rise in 25(OH)D levels in those infants randomized to the 400 IU vitamin D_3_/one drop/day regimen. Of the 33 infants who have completed the study, the mean levels increased from 16.0 ± 9.3 ng/mL (baseline; range 1.0–40.8 ng/mL; median 13.4) to 43.6 ± 14.1 ng/mL (range 18.2–69.7) at 4 months and remained relatively unchanged at month 7 : 42.5 ± 12.1 ng/mL (range 18.9–67.2). As defined by circulating 25(OH)D levels <20 ng/mL, of the 33 infants, 24 (72.7%) had evidence of deficiency at one month of age. The change in values between 1 and 4 months and 1 and 7 months was statistically significant (*P* ≤ .0001). As predicted, there were no statistically significant differences between months 4 and 7 (*P* = .66). Repeated measures ANOVA indicated overall significance (*P* < .0001). The difference was unaffected by seasonal variation (Table 2). There was no toxicity observed in the infants based on serum calcium, phosphorus, and creatinine levels. In addition, there were no adverse health effects attributed to vitamin D supplementation.

When examining the 400 IU vitamin D/day dose on a weight basis, there was a significant change in IU/kg over the time for the 33 infants who completed the study: at visit 1, the infants were receiving 88.9 ± 10.5 IU/kg; at visit 4, they were receiving 59.7 ± 6.6 IU/kg; and at visit 7, they were receiving 50.5 ± 6.0 IU/kg (*P* < .0001). Despite the decrease in dose on a per kilogram basis, as mentioned earlier, the infant mean circulating 25(OH)D levels were not significantly different between visit 4 and 7. After controlling for season and skin pigmentation changes due to sunlight exposure, results remained significant.

## 4. Discussion

Because the preliminary results of this arm of the trial are important for decision-making in the current clinical environment, we report here the interim results of an ongoing vitamin D supplementation trial of lactating women and their infants. In this ongoing study of lactating women and their infants who were randomized to one of three treatment groups, we sought to determine whether the one drop per day of an oil emulsion vitamin D_3_ preparation is effective in raising the infants' levels to the desired target of >30 ng/mL (~ 80 nmol/L). Infant's 25(OH)D levels consistently and significantly increased to a plateau by three months of therapy on the daily oil emulsion preparation dispensed as one drop per day to deliver 400 IU. Overall, the infant's circulating 25(OH)D levels increased 37% above baseline. There were no adverse events associated with the prescribed vitamin D supplementation. While not powered to assess safety at this juncture, it is important to note that, thus far, there have been no increased rates of infection in the infants or adverse health effects that could be attributed to vitamin D supplementation in this cohort.

Another important finding in this study is that the majority of infants enrolled in this cohort of exclusively breastfed infants had vitamin D deficiency (defined as circulating 25(OH)D levels <20 ng/mL) at one month of age. Infant vitamin D levels in exclusively breastfed infants reflect maternal vitamin D status, and given that the majority of the mothers in this cohort present with circulating 25(OH)D levels <30 ng/mL, it is not surprising that the infants who are almost solely dependent on the mother for their vitamin D have corresponding deficiency. Until it can be determined what amount of vitamin D supplementation in mothers will effectively and safely increase and/or maintain the vitamin D status in her breastfed infant, we must rely on vitamin D supplementation of the infant to prevent deficiency. Thus, our findings in this study and our earlier studies [4, 32] support the recent AAP recommendation of starting vitamin D supplementation in all breastfed infants within the first few days after delivery [26].

Concerns about dosing infants with any medication, particularly as a single drop, are warranted. The greatest risk appears when a parent is not instructed on how to give a medication or does not receive the full instruction on proper dispensing. We have found in our study of lactating women that demonstrating how to give the drop is the best method of teaching proper dispensing. Mothers are asked to demonstrate how to give the drop prior to leaving the research outpatient clinic, and this method has been found to be effective.

Our findings support the premise that an oil emulsion delivered as a single drop is well-tolerated and absorbed with a consistent increase in circulating 25(OH)D on the vitamin D supplementation during a three-month interval that was sustained during the next three-month interval. While prescription of a vitamin or medication and changes in blood levels of that vitamin or medication, in this case, vitamin D does not prove direct causality, the change in circulating 25(OH)D status was independent of season. In addition, despite the normal growth of the infants during this six-month period, on a per kilogram basis, the 400 IU/day dose was adequate in helping the infant maintain adequate vitamin D status during the six-month study period.

Limitations of this study include its small sample size. This study is designed to enroll 126 subjects into the control arm. To date, 54 subjects have been enrolled and 33 have completed the study. We acknowledge that this interim report is based on the first 33 to have completed this ongoing trial and that while this represents ~ 61% of the 54 enrolled and 26% of the total projected cohort, we believe that the results are generalizable since timing of enrollment is random. There would be no underlying systematic bias that would suggest that the results of the first 33 subjects would differ from the middle 33 or the last 33, especially when seasonality is controlled as a potential confounder, which was done. 

Another limitation of this study is the lack of a placebo control group. While a group of infants that received placebo would allow for more refined statistical analyses, it is deemed unethical by the investigators and the IRB not to supplement the infants with what is now the standard of care for vitamin D supplementation of infants and children in both the U.S. and Canada (see AAP statement 2008 [5] and Canadian Paediatric Society statement 2007 [33]). The direct effect of the vitamin D supplementation is supported by the following: in this trial, of the mothers who were randomized to receive 2400 IU vitamin D/day, there was insufficient transfer of vitamin D in their milk and 31% of those breastfed infants required open label vitamin D supplementation at the 4th month visit compared with 6% in the control group and 5% in the 6400 IU vitamin D supplementation group. As a result, the 2400 IU arm of the study was stopped in February 2008 (unpublished data). Clearly, the 400 IU/day group of infants (presented here) had improved vitamin D status based on their therapy that was not seen in the 2400 IU group of infants, who were receiving placebo and whose only source of vitamin D was maternal breast milk that has significantly lower levels than that of the 6400 IU group. 

In summary, a single drop oil emulsion vitamin D preparation appears to be an effective method to deliver vitamin D_3_ to a breastfed infant. The safety of this regimen beyond this small cohort remains to be determined. Further evaluation of this method also is warranted in infants who have underlying disease states.

## Figures and Tables

**Table 1:** Infant weight, vitamin D status, and dosage per body weight.

Variable	Visit 1 (*n* = 33)	Visit 4 (*n* = 33)	Visit 7 (*n* = 33)
Infant wt (mean ± S.D.)	4.6 ± 0.52 kg	6.8 ± 0.75 kg	8.0 ± .98 kg
Total circulating 25(OH)D (ng/mL)	16.0 ± 9.3	43.6 ± 14.1	42.5 ± 12.1
IU vitamin D/body wt (kg)	88.9 ± 10.5	59.7 ± 6.6	50.5 ± 6.0

**Table 2:** Circulating 25(OH)D (ng/mL) at visits 1, 4, and 7 by season. Sunny months at latitude 32°N based on ultraviolet light indices occur from April through October. Relative nonsunny months comparatively occur from November through March.

	25(OH)D (ng/mL)	25(OH)D (ng/mL)	25(OH)D (ng/mL)
	Visit 1	Visit 4	Visit 7
Sunny months	18.5	46.0	48.0
Nonsunny months	13.2	42.1	37.3
